# Combined radiation injury and its impacts on radiation countermeasures and biodosimetry

**DOI:** 10.1080/09553002.2023.2188933

**Published:** 2023-03-22

**Authors:** Juliann G. Kiang, William F. Blakely

**Affiliations:** a Radiation Combined Injury Program, Scientific Research Department, Armed Forces Radiobiology Research Institute, Uniformed Services University of the Health Sciences, Bethesda, MD, USA;; b Department of Pharmacology and Molecular Therapeutics, Uniformed Services University of the Health Sciences, Bethesda, MD, USA;; c Department of Medicine, Uniformed Services University of the Health Sciences, Bethesda, MD, USA;; d Biodosimetry Program, Scientific Research Department, Armed Forces Radiobiology Research Institute, Uniformed Services University of the Health Sciences, Bethesda, MD, USA;; e Department of Preventive Medicine and Biostatistics, Uniformed Services University of the Health Sciences, Bethesda, MD, USA

**Keywords:** Combined radiation injury, wounding, medical countermeasures, mechanisms, acute radiation syndrome, biodosimetry

## Abstract

**Purpose:**

Preparedness for medical responses to major radiation accidents and the increasing threat of nuclear warfare worldwide necessitates an understanding of the complexity of combined radiation injury (CI) and identifying drugs to treat CI is inevitably critical. The vital sign and survival after CI were presented. The molecular mechanisms, such as microRNA pathways, NF-*κ*B-iNOS-IL-18 pathway, C3 production, the AKT-MAPK cross-talk, and TLR/MMP increases, underlying CI in relation to organ injury and mortality were analyzed. At present, no FDA-approved drug to protect, mitigate, or treat CI is available. The development of CI-specific medical countermeasures was reviewed. Because of the worsened acute radiation syndrome resulting from CI, diagnostic triage can be problematic. Therefore, biodosimetry and CI are bundled together with the need to establish effective triage methods with CI.

**Conclusions:**

CI mouse model studies at AFRRI are reviewed addressing molecular responses, findings from medical countermeasures, and a proposed plasma proteomic biodosimetry approach based on a panel of radiation-responsive biomarkers (i.e., CD27, Flt-3L, GM-CSF, CD45, IL-12, TPO) negligibly influenced by wounding in an algorithm used for dose predictions is described.

## Introduction

Radiation exposure events on a large scale have always found that victims exposed to radiation are also often suffering from other trauma including hemorrhage, blast, burns or wounds. Combined radiation injuries (CI) were documented at Hiroshima and Nagasaki, Japan, where 60–70% of radiation victims concurrently received thermal burn injury (Iijima 1982; Kishi 2000), while there are reports indicating victims with approximately 60% radiation alone, 35% 2-injuries, and 5% 3-injuries (Table 1). At the Chernobyl reactor meltdown, 10% of 237 victims were exposed to radiation and thermal burns together (Barabanova 2006). Therefore, combined radiation injury is an important area requiring attention, at least with a similar degree as radiation injury alone.

## Combined radiation injury

In animal models of combined radiation injury (CI), there are rats (Alpen and Sheline 1954; Valeriote and Baker 1964), guinea pigs (Korlof 1956), dogs (Brooks et al. 1952), and swine (Baxter et al. 1953) with burns and wounds; they usually rise mortality after otherwise non-lethal radiation exposures. In 1970s, Armed Forces Radiobiology Research Institute (AFRRI) began CI investigations. In mice, radiation exposure followed by burns or other wounds further reduced survival compared to burns alone, wounds alone or radiation exposure alone (Ledney et al. 1992; Kiang and Ledney 2013), and radiation delays wound healing times (Ledney et al. 1981). Subsequently, CI resulted in acute suppression of myeloid, inhibition of the immune system, fluid imbalance, macro/microcirculation failure, massive cellular damage and death, and vital organ dysfunctions. Then, multiple organ dysfunction syndrome (MODS) occurs, which is the most frequent cause of death after CI (Koenig et al. 2005; Lausevic et al. 2008; Zou et al. 2008; Kiang and Olabisi 2019).

In addition to penetrating skin wounds (Kiang et al. 2010b) and hemorrhage (Kiang et al. 2015, 2017a), CI with skin burn (Ledney and Elliott 2010; Kiang et al. 2014b) and infection (Brook et al. 2001; Elliott et al. 2002) were established at AFRRI. The radiation dose modified factors (DMF) for radiation combined with skin wound and hemorrhage were 1.08 (Kiang et al. 2010b) and 1.04 (Kiang et al. 2015), respectively, whereas the DMF of radiation in combination with burn was between wound and hemorrhage (Kiang, unpublished data). Radiation combined with infection was toxic but the DMF was not yet determined. Besides worsened survival, body weight loss and delayed wound healing, CI with radiation plus wound (Swift et al. 2015a) or plus hemorrhage (Kiang et al. 2015) appeared to amplify and prolong skeletal tissue loss.

The first study on molecular mechanisms underlying CI (Kiang et al. 2010b) showed that radiation followed by skin wounding immensely increased a) inducible nitric oxide synthase (iNOS), b) cytokine/chemokine concentrations in serum, and c) systemic bacterial infection in liver and heart significantly more than radiation alone (Kiang, et al. 2010b; Kiang, et al. 2020). Their details are described as follows.

The iNOS in these B6D2F1 female mice exposed to CI was largely increased, which was mediated by elevated nuclear factor-keppaB (NF-jB) and nuclear factor-interleukin 6 (NF-IL6) (Kiang et al. 2010b). NF-IL6 was usually stimulated by IL-6 which was increased by radiation. Moreover, the increased iNOS triggered the breakage of the tight junction of the gut barrier as measured by the reduction of Claudin-2, as a well-accepted biomarker for tight junction (Kiang et al. 2020). The magnified increases in systematic bacteria after CI included *Enterococcus, Bacillus, Lactobacillus, and Staphylococcus*, while systemic bacteria after radiation injury (RI) were *Enterococcus* and *Staphylococcus* (Kiang et al. 2010b), which was confirmed by measuring the bacterial DNA and lipopolysaccharide (LPS) concentrations in the liver (Kiang et al. 2020). The onset time of this sepsis was on day 3 after CI and on day 6 after RI (Kiang et al. 2010b). In CD2F1 male mice exposed to radiation followed by 20% hemorrhage, CI mice were detected with *Streptococcus sanguinis* and *Sphingomonas paucimobilis* and RI mice were with *Proteus mirabilis* (Kiang et al. 2017a). The differences in bacterium found after CI could be due to (i) different strains of mice studied, (ii) different sex of mice used, and (iii) different traumas applied, namely, wounding verse hemorrhage.

Using 23 cytokine/chemokine multiplex luminex, we found radiation increased IL-6, IL-10, KC, G-CSF, and MCP-1. In contrast, CI significantly further increased these and many others including IL-1*β*, IL-6, IL-9, IL-10, IL-13, KC, G-CSF, Eotaxin, IFN-γ, MCP-1, MIP-1*α*, and MIP-1*β* (Kiang et al. 2010b). In mice exposed to radiation followed by 20% hemorrhage, RI increased IL-1*β*, IL-2, IL-12p40, IL-17A, G-CSF, IFN-γ, KC, MCP-1, IL-15, and IL-18, whereas CI increased IL-1*α*, IL-1*β*, IL-3, IL-6, IL-9, IL-10, IL-12p40, IL-12p70, IL-13, IL-17A, G-CSF, IFN-γ, KC, MCP-1, MIP-1*β*, TNF-*α*, Il-15 and IL-18 (Kiang et al. 2017a).

Radiation with skin wound (CI-wound) significantly increased p21, Bax, Bcl-2, Bax/Bcl-2 ratios, DDB2, Cadd45*α*, and TERT (Kiang et al. 2010a). Additionally, altered Cdh6, Itga7, Mmp-2, −3, −7, −9, −10, −11, −13, Timp3, Timp4, TLR-1, −2, −4, −6, −7, −8, −9, and myd88 (Kiang and Olabisi, 2019). AKT (pro-survival) and MAPK (anti-survival) were deactivated and activated, respectively, by RI and CI (Kiang et al 2020). It is evident that CI with mixed-field (neutron + gamma) radiation followed by skin wound synergistically increased corticosterone, decreased C-reactive protein (CRP), increased complement component 3 (C3), and prostaglandin E_2_ (PGE_2_) and decreased immunoglobulin (IgM) (Kiang and Ledney 2013). CI with Co-60 radiation followed by 20% hemorrhage increased corticosterone, decreased CRP, and increased both C3 and Flt-3 ligands (Kiang et al. 2017a).

It is evident that skin wounds following high-dose radiation exposure amplified and prolonged skeletal tissue loss indicated by biomarkers including transient TRAP 5b increases, decreased osteocalcin, and increased sclerostin (Swift et al. 2015a). Radiation followed by hemorrhage resulted in trabecular bone loss as indicated by increased TRAP 5b, decreased osteocalcin, and increased sclerostin as well (Swift et al. 2015b).

**Figure 1.**
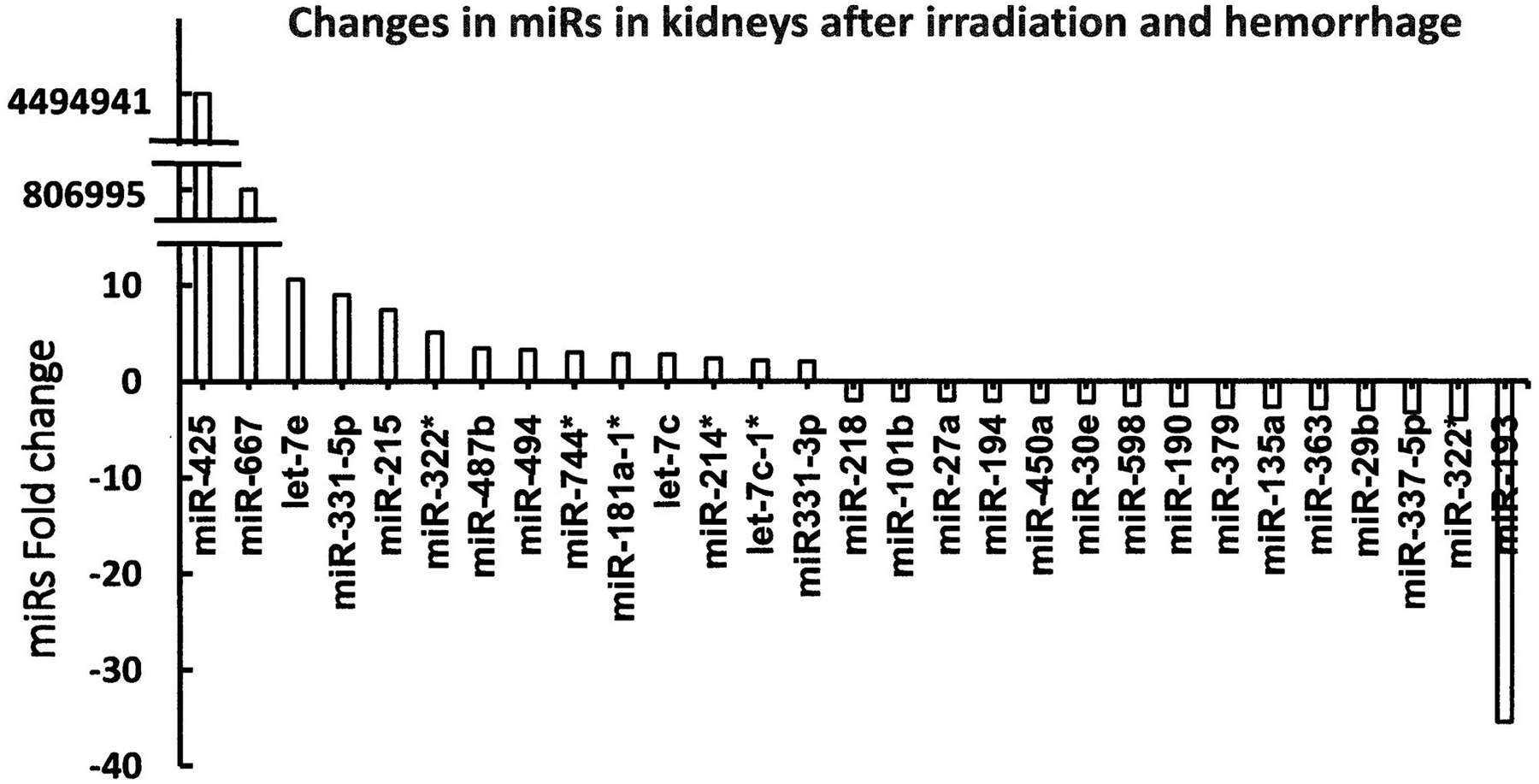
CI increases microRNAs in kidneys of mice exposed to radiation followed by hemorrhage. CD2F1 male mice received 8.75 Gy ^60^Co followed by 20% bleeding (Kiang et al. 2017a). Kidneys were collected on day 1 after CI (N = 6 per group). miR; microRNA. These results show changes reflecting greater than 2-fold changes (either increased or decreased) fold changes compared to the 0 Gy group.

**Figure 2.**
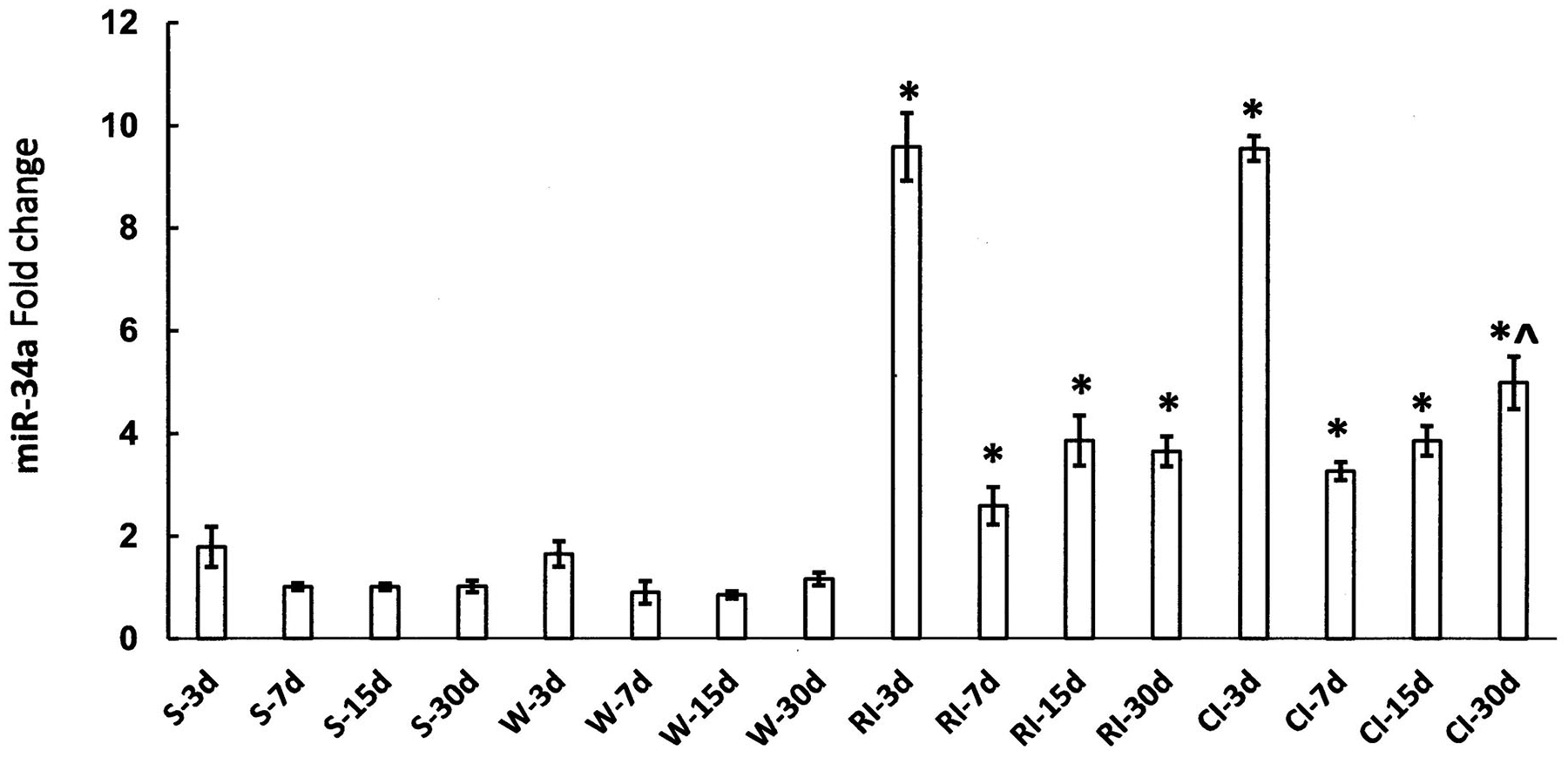
CI-induced miR-34a increases are not different from RI at early time points. B6D2F1 female mice received 9.5 Gy ^60^Co alone or followed by 15% total body surface area wound trauma (Kiang et al. 2010b). Ileums were collected on days 3, 7, 15 and 30 after RI or CI. No differences between the RI-induced increases in miR-34a and the CI-induced increases in miR-34a were found on days 3, 7, and 15. On day 30, the CI-induced increases were significantly greater than the RI-induced increases (N = 4 per group). *p < .05 vs. respective sham or wound alone; ^p<.05 vs. R-30d using T-Test. miR: microRNA (Kiang et al. 2022 for Materials and Methods). S: Sham; W: 15% total body surface wound; RI: 9.5 Gy; CI: 9.5 Gy + 15% total body surface wound. These results show changes compared to the sham group.

CI with hemorrhage following high-dose radiation exacerbates CI-induced EPO and HIF-1*α* due to increased NF-*κ*B in the kidney. The inflammation-associated microRNA (miR) expression in the kidney was also altered. Among 29 miRs on day 1 after RCI (Figure 1), 14 were significantly increased and 15 were significantly decreased. Let-7e, miR-29b, miR-30e, miR-27a, miR-32 and miR-135 were associated with NF-*κ*B, HIF-1*α*, and EPO (Kiang et al. 2017a). However, CI with skin wound following high-dose radiation increased miR-34a to a similar magnitude as RI at early times but not on day 30 (Figure 2). MiR-34a was inhibited by G-CSF. MiR-34a decreased Bcl-2 and increased Bax, whereas G-CSF increased Bcl-2. Therefore, the balance between G-CSF and miR-34a should be critical for cell survival or death (Kiang et al. 2022). It should be kept in mind that FMS-like tyrosine kinase 3 ligand (flt-3L, a bone marrow dysplasia marker) was increased by RI and CI, but the RI-induced increases and CI-induced increases were similar (Jiao et al. 2010; Kiang et al. 2017a). In contrast, citrulline (produced by intestinal epithelial cells) was increased by RI and CI, but the CI-induced decreases were more than RI-induced decreases, confirming the CI-induced GI damage was more than the RI-induced GI damage (Kiang et al. 2020).

ATP is critical for biomolecular phosphorylation. Our laboratories reported that CI significantly induced ATP depletion in GI, pancreas, spleen, and kidney due to decreases in pyruvate dehydrogenase and increases in pyruvate dehydrogenase kinase 1 (Swift et al. 2015a), decreases in NRF1, NRF2 in cytosol, complexes 1–5 in mitochondria (Kiang et al. 2019) and mitochondrial remodeling (Gorbunov et al. 2015).

Table 2 lists the molecules changed by CI with RI plus wounding, burning, or hemorrhage. CI with RI + wound or RI + hemorrhage was studied extensively and displayed similar changes, whereas studies of CI with RI + burning were limited and required further exploration before a true comparison can be achieved among these 3 CIs. With current comparisons among the three combinations, increases in C3 and corticosterone and decreases in ATP production were commonly found.

## Countermeasures tested for CI

FDA has approved Neupogen (Amgen Inc 2015a), Neulasta (Amgen Inc2015b), Nplate (Amgen Inc 2021), and Leukine (Safoni-Aventis 2018). They are approved as mitigators to treat hematopoietic syndrome. Despite their efficacy to mitigate radiation-induced hematopoietic acute radiation syndrome, none of them is efficacious for treating CI.

To determine whether a drug is qualified as a medical countermeasure, 30-day survival, body weight loss, and wound healing time are the criteria when the animal is exposed to radiation at LD50/30. Many drugs were tested. Many of them failed to improve survival, mainly due to neither mitigation in body weight loss nor acceleration of wound healing. However, Table 3 lists that treatment with single therapy including gentamicin, Silvadene, WR-151337 (Ledney and Elliott 2010), Alxn4100TPO (Kiang et al. 2017b), bone marrow transplant (Ledney and Elliott 2010), mesenchymal stem cells (Kiang and Gorbunov 2014), Ghrelin (Kiang et al. 2014b; Kiang, Anderson, et al. 2018, 2020), and Ciprofloxacin (Fukumoto et al. 2014; Kiang and Fukumoto 2014). Table 4 lists combined therapy such as 5-S-TDCM plus Gentamicin (Ledney and Elliott 2010), Neulasta plus Alxn4100TPO (Kiang et al. 2017b) and Neulasta plus L-citrulline (Wang et al. 2021) resulted in increases in survival after CI.

## Single therapy

### • Effects of Gentamicin on CI

Gentamicin (0.1% in Garamycin cream) is one of antibiotics. C3H/HeN female mice received gentamicin topically covering the entire wounded area 4 hr after CI and thereafter once daily for 9 days. The mixed-field (67% neutron + 33% gamma) irradiation increased LD50/30 from 3.13 Gy to 4.26 Gy with a dose reduction factor (DRF) of 1.36. No body weight loss and wound healing measurements were conducted (Ledney and Elliott 2010).

### • Effects of silvadene on CI

Silvadene (1% silver sulfadiazine) is one of the antibiotics. C3H/HeN female mice received silvadene topically covering the entire wounded area 4 hr after CI and thereafter once daily for 9 days. The mixed-field (67% neutron + 33% gamma) irradiation increased L50/30 from 3.13 Gy to 3.74 Gy with DRF of 1.16. No body weight loss and wound healing measurements were conducted (Ledney and Elliott 2010).

### • Effects of WR-151327 on CI

WR-151327 is S-3-(3-methylaminopropylamino) propylthiophosphorothioic acid. C3H/HeN female mice received this drug (200 mg/kg, i.p.) 30 min before RCI. The mixed-field (67% neutron + 33% gamma) irradiation increased CI LD50/30 from 3.75 Gy to 4.55 Gy with DRF of 1.22. The pure gamma irradiation increased CI LD50/30 from 7.60 to 11.50 with DRF of 1.51. No body weight loss and wound healing measurements were conducted (Ledney and Elliott 2010).

### • Effects of Alxn4100TPO on CI

Alxn4100TPO is a thrombopoietin (TPO) receptor agonist. It was subcutaneously administered with 1 mg/kg s.c. 24 hr to B6D2F1 female mice after CI. It significantly attenuated body weight loss and increased 30-day survival by 20%, but failed to accelerate wound healing. It significantly increased platelet counts (Kiang et al. 2017c). The results suggest that failure in wound healing acceleration limits its efficacy in improving survival after CI.

### • Effects of bone marrow transplant on CI

Whole bone marrow collected from femurs (1 × 10^4^ to 20 × 10^6^ cells per mouse in 0.5 ml RPMI-1640 media) was intravenously delivered to C3H3 female mice within 2 hr after CI. 100% survival was observed. Similar observations were also obtained in mice exposed to RI, but the bone marrow cells required were 10 times lower than CI (Ledney and Elliott 2010). Neither body weight loss nor wound healing rates were reported.

### • Effects of mesenchymal stem cells on CI

B6D2F1 female mice were administered with bone marrow-derived mesenchymal stem cells extracted from femurs at 3×10^6^ cells in 0.4 ml DMEM through the tail vein 24 hr after LD70/30 CI. The survival was increased by 30% above the vehicle-treated group. The body weight loss was mitigated. The wound was healed 7 days earlier than the vehicle-treated group. The histopathology with H&E staining appeared to increase cellularity (Kiang and Gorbunov 2014).

### • Effects of Ghrelin on CI

Ghrelin, 28 amino acids, is released from the stomach during hunger and triggers appetite through the thalamus. B6D2F1 female mice were administered with ghrelin at 113 mg/kg i.v. 24 hr, 48 hr, 72 hr after CI. This hunger-stimulating peptide therapy increased survival, mitigated body-weight loss, accelerated wound healing, and numbers of neutrophils, lymphocytes, and platelets and ameliorated bone-marrow cell depletion (Kiang et al. 2014b). Additionally, this therapy appeared to effectively inhibit RCI-induced brain surface hemorrhage and intracranial hemorrhage (Gorbunov and Kiang 2017; Kiang et al. 2019).

This laboratory reported that Ghrelin enabled to mitigate the of CI-induced bone marrow injury and splenocytopenia by sustaining G-CSF and KC increases in circulation (Kiang et al. 2018). Additionally, Ghrelin therapy mitigated CI-induced increases in IL-1*β*, IL-6, IL-17A, IL-18, KC and TNF-*α* in serum but sustained G-CSF, KC and MIP-1*α*increases in GI. Moreover, Ghrelin increased AKT activation and ERK activation and suppressed JNK activation, caspase-3 activation, NF-*κ*B activation, iNOS, and BAX. In turn, the tight junction in GI was repaired so to mitigate bacterial translocation from GI lumen into the tissue (Kiang et al. 2020), suggesting Ghrelin improves survival mediated by organ repairs such as bone marrow, GI, and brain repair, or mitigation of hematopoietic ARS, GI ARS, and Neurovascular ARS.

### • Effects of Ciprofloxacin on CI

Ciprofloxacin, Ciprofloxacin is an FDA-approved fluoroquinolone (FQ), which is widely used as an antimicrobial. Ciprofloxacin has been included in the Strategic National Stockpile, which is maintained by the U.S. Department of Health and Human Services, to control bacterial infection during a national emergency such as a nuclear detonation or other radiological incidents. Besides the antimicrobial activity, several groups reported immunomodulatory effects that Ciprofloxacin exerts in rodent models and human clinical trials (Dalhoff and Shalit 2003; Dalhoff 2005), improving a wide spectrum of conditions including thrombocytopenia (Shoenfeld et al. 1998; Savion et al. 2000; Blank et al. 1998), Crohn’s disease (Stein and Hanauer 1999; Rath et al. 2001), rheumatoid arthritis (Breban et al. 1992; Lewis and Keft 1995) and chemotherapy-induced neutropenia (Freifeld et al. 1999). Most importantly, oral administration with Ciprofloxacin at 90 mg/kg, 2 hr after CI and once daily thereafter for 21 days, significantly increased survival, mitigated body weight loss and accelerated skin-wound healing (Fukumoto et al. 2014; Kiang and Fukumoto 2014). Ciprofloxacin improved recovery of bone marrow cellularity (Fukumoto et al. 2014), enhanced stress erythropoiesis in the spleen that was stimulated by circulating IL-3 increases (Fukumoto et al. 2014; Kiang and Fukumoto 2014), and mitigated ATP loss in GI, pancreas, spleen, and kidney via preservation of pyruvate dehydrogenase and inhibition of pyruvate dehydrogenase kinase 1 (Swift et al. 2015a). Furthermore, Ciprofloxacin acted as a radio-sensitizer to tumor cells and a radio-protectant for normal cells via differential effects on γ-H2AX formation, p53 phosphorylation and Bcl-2 production (Kiang et al. 2014c). Repurposing Ciprofloxacin in this regard will be beneficial to treat CI in the near future due to the increasing threat of using nuclear weapons worldwide.

## Combined therapy

Combined therapies of 5-S-TDCM plus Gentamicin (Ledney and Elliott 2010), Neulasta plus Alxn4100TPO (Kiang et al. 2017b), or Neulasta plus L-Citrulline (Wang et al. 2021) are effective in increasing the survival after RCI, likely by enhancing the survival of the hematopoietic stem/progenitor cells, GI repair, or accelerating recovery of cutaneous wounds. Moreover, co-therapy of Neulasta with Ghrelin mitigated the radiation-induced brain hemorrhage (Kiang et al. 2019), even though treatment with Ghrelin alone showed to significantly diminish the brain hemorrhage induced by radiation combined with burn trauma (Gorbunov and Kiang 2017).

### • Effects of S-TDCM plus Gentamicin on CI

Synthetic trehalose dicorynomycolate (S-TDCM), a nonspecific immuno- and hematopoietic modulator. C3H3 female mice were exposed to 8 Gy RCI and immediately received S-TDCM (200 mg/kg, i.p.) with topical gentamicin. This combined therapy increased 30-day animal survival. There were no reports on body weight loss and wound healing impacted by this combined therapy (Ledney and Elliott 2010).

### • Effects of Neulasta plus Alxn4100TPO on CI

Alxn4100TPO is a TPO receptor agonist and known to increase platelets and survival after RCI (Kiang et al. 2017b). B6D2F1 female mice were injected with Neulasta^™^ at 1 mg/kg, subcutaneously on days 1, 8 and 15 after RCI and Alxn4100TPO (1 g/kg), once 4 hr after RCI. The results suggest that combined treatment with Neulasta and Alxn4100TPO is effective for mitigating the effects of both radiation alone and in combination with wound injury. Each individual drug alone or the combined treatment resulted in the mitigation of body weight loss, WBC depletion, and platelet depletion, but did not accelerate wound healing (Kiang et al. 2017b).

### • Effects of Neulasta plus L-Citrulline on CI

L-citrulline is a neutral alpha-amino acid shown to improve vascular endothelial function in cardiovascular diseases (Romero et al. 2006; Matsuo et al. 2017). B6D2F1 female mice were injected with Neulasta at 1 mg/kg, subcutaneously on days 1, 8 and 15 after RCI and L-citrulline (1 g/kg), once daily from day 1 to day 21 after CI. The combined therapy significantly improved the 30-day survival by 27% above the vehicle controls. This co-therapy significantly mitigated body weight loss increased bone marrow stem and progenitor cell clonogenicity, and accelerated recovery from intestinal injury. Although treatment with L-citrulline alone accelerated skin wound healing after CI, the co-therapy did not (Wang et al. 2021).

Data from our laboratory and others suggest that sex disparity to radiation sensitivity is present. Females were found to be more resistant to radiation than males (Kiang et al. 2022; Krukowski et al. 2018). In addition, other confounding factors including age, hypertension, diabetes, and high cholesterol should be addressed. However, it is imminent and imperative to explore whether the sex disparity is also present in the efficacy of radiation medical countermeasures for RI or CI.

Among all FDA-approved drugs and non-FDA-approved candidates, combined therapy with mesenchymal stem cells (Kiang and Gorbunov 2014) and Neulasta (Kiang et al. 2014a) should stand a great chance to significantly increase survival after CI, because whole bone marrow (containing pregenital stem cells and mesenchymal stem cells) transplantation displayed 100% survival after lethal CI (Ledney and Elliott. 2010), treatment with mesenchymal stem cells showed survival improvement by 30% after lethal CI (Kiang and Gorbunov 2014), and Neulasta demonstrated to mobilize lymphocytes from bone marrow to peripheral blood (Kiang et al. 2014a). In this case, if Neulasta can enhance mesenchymal stem cell capability to further save more lives after CI, then fewer numbers of mesenchymal stem cells are needed, thereby, leading to fewer chances to develop lung fibrosis later. However, there is a drawback to using mesenchymal stem cells, because medical assistance is required that is not feasible under a mass casualty scenario. In addition, the quality control and/or safety use of mesenchymal stem cells are not officially regulated yet.

## CI and biodosimetry

**Figure 3.**
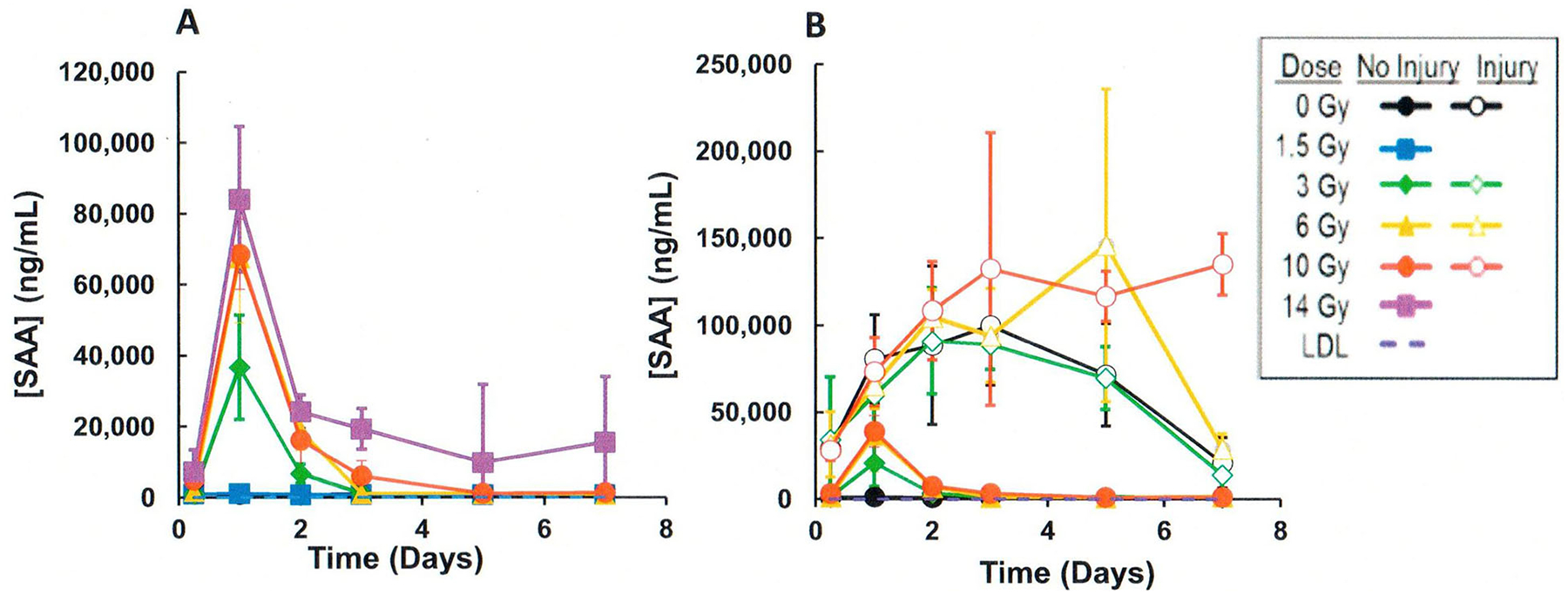
Comparison of effects of radiation vs CI (radiation plus wounding) on plasma serum amyloid A (SAA) in mouse radiation model. (A) Radiation exposure, (B) CI (radiation plus wounding) exposure. Symbols represent the mean and bar the standard error of the mean. Comparison of radiation vs radiation plus wounding samples showed significant differences using the T-test (p < .05) (Supplementary Table 1).

**Figure 4.**
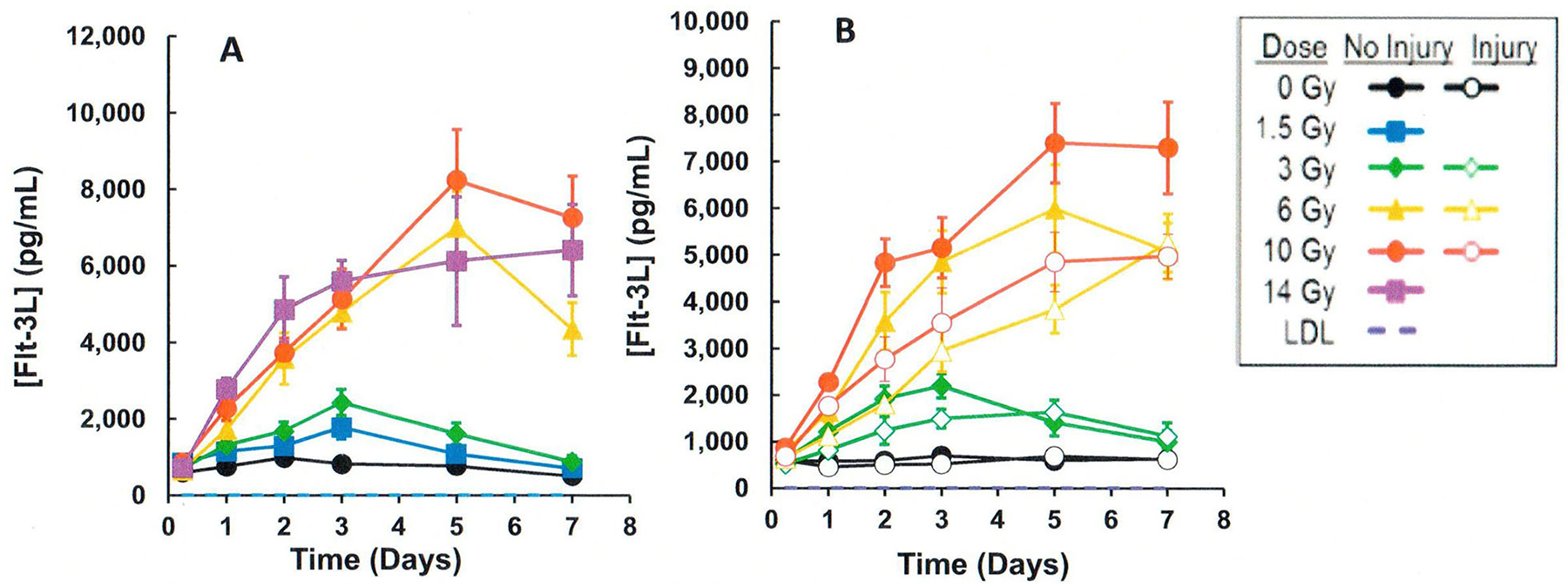
Comparison of effects of radiation vs. CI (radiation plus wounding) on plasma Fms-like tyrosine kinase 3 ligands (Flt-3L) in mouse radiation model. (A) Radiation exposure, (B) CI (radiation plus wounding) exposure. Symbols represent the mean and bar the standard error of the mean. Comparison of radiation vs radiation plus wounding samples showed either significant differences using the T-test (p < .05) or were less than ~2-fold different (Supplementary Table 1).

**Figure 5.**
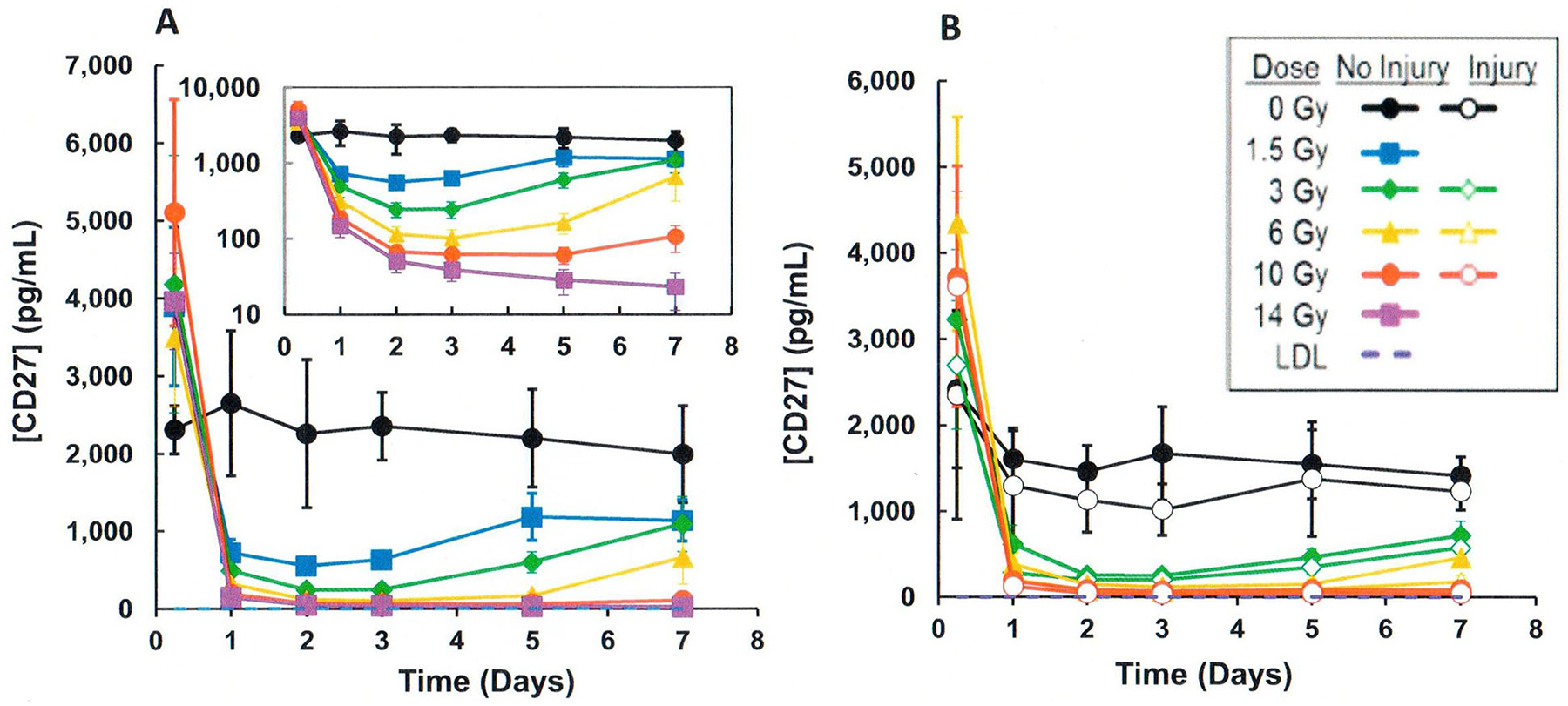
Comparison of effects of radiation vs. CI (radiation plus wounding) on plasma cluster differentiation 27 (CD27; lymphocyte surface biomarker) in mouse radiation model. Materials and methods as described in Figure 3 legend. (A) Radiation exposure, (B) CI (radiation plus wounding) exposure. Symbols represent the mean and bar the standard error of the mean. Comparison of radiation vs radiation plus wounding samples showed either significant differences using the T-test (p < .05) or were less than ~2-fold different (Supplementary Table 1).

Studies were performed to evaluate the effects of CI (radiation plus wounding) on the use of plasma proteomic biomarkers to assess radiation dose (Ossetrova and Blakely 2009; Sigal et al. 2013; Blakely et al. 2014; Ossetrova et al. 2014). A mouse study using female B6D2F1/J mice (N = 8 per dose and time point) was performed comparing a panel of candidate proteomic plasma radiation-responsive biomarkers after exposure to radiation compared to radiation and wounding involving a dorsal puncture over 15% body surface (CI) within 1–2 hr after radiation exposure. Blood was biosampled at various times after exposure to ^60^Co gamma rays (0.6 Gy/min) to a dose spanning from 0 to 10 Gy. Several radiation-response biomarkers (i.e. serum amyloid A or SAA, G-CSF, CD27) showed large wounding injury effects compared with the radiation-only response. For example, Figure 3 illustrates the response when measuring SAA, which illustrates significant (p < .05) elevations of SAA levels by wounding at equivalent radiation exposures. In contrast, several blood plasma proteomic biomarkers (i.e., Fms-like tyrosine kinase 3 ligand or Flt-3L, GM-CSF, TPO, EPO, IL-5, and CD27) were negligibly affected by wounding. For example Figures 4 and 5 illustrate the response when measuring Flt-3L (Figure 4) and CD27 (Figure 5), where RI and wounding (CI) showed either minor changes (i.e., fold differences less than ~2) or no significant differences (p < .05); see Supplementary Table 1 for data and statistical analysis. CI did not worsen or diminish radiation-induced increases in Flt-3 ligand (Jiao et al. 2009; Kiang et al. 2017b) or miR-34a at early time points before day 7 after CI (Figure 2).

**Figure 6.**
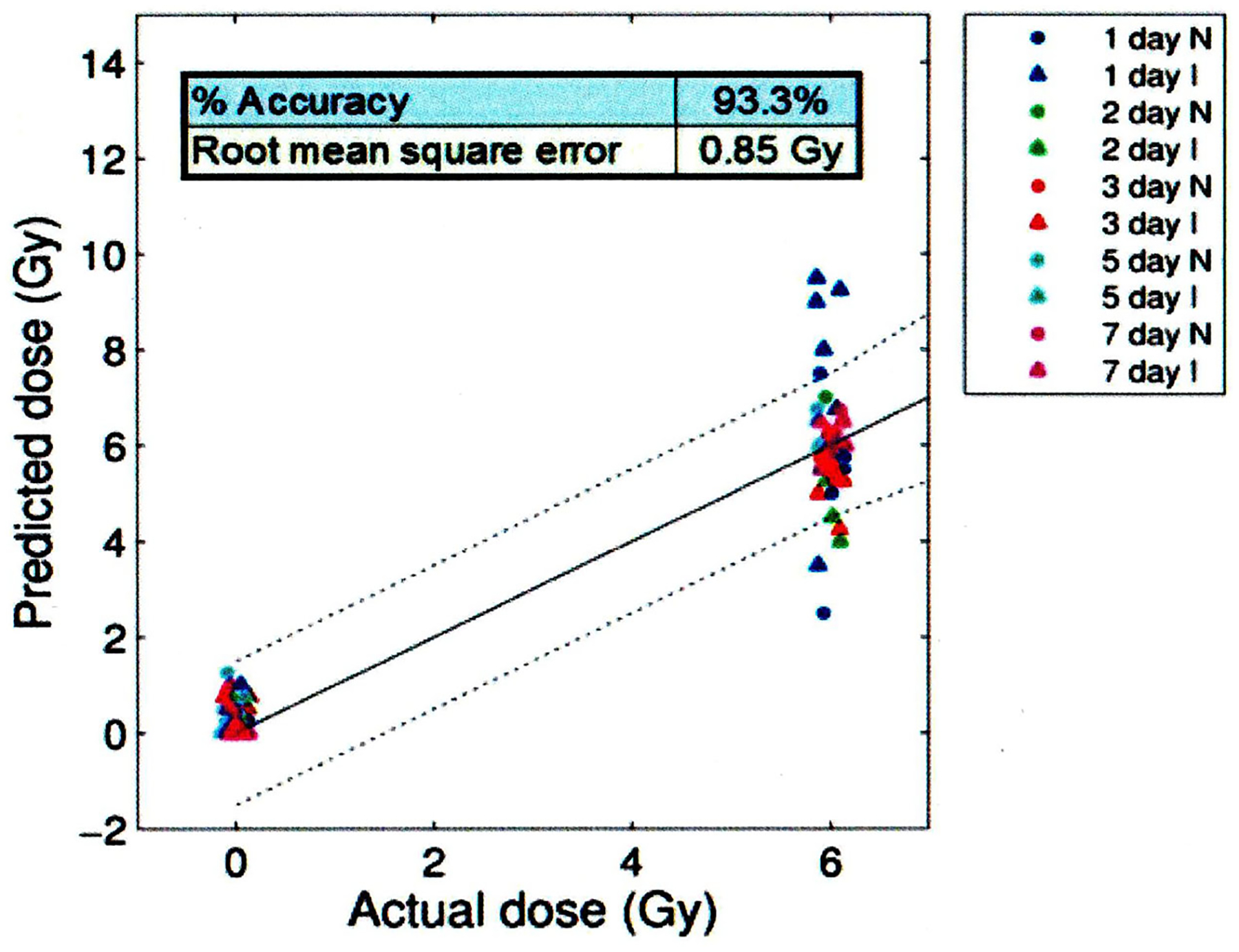
Effect of CI (radiation plus wounding) on radiation dose prediction accuracy based on an algorithm using a panel (i.e., CD27, Flt-3L, GM-CSF, CD45, IL-12, TPO) of plasma biomarkers negligibly affect by combined radiation and wounding. Mice received 0 and 6 Gy with or without skin wounding. Symbols represent the results from individual animals at the designated time points after irradiation. Time points within the dashed lines meet the established dose prediction accuracy criteria. CD27: cluster differentiation 27; Flt-3L: Fms-like tyrosine kinase 3 ligand; GM-CSF: granulocyte-macrophage-colony stimulating factor; CD45: cluster differentiation 45; IL-12: interleukin 12; TPO: thrombopoietin; N: not injured; I: injured.

A multivariate algorithm was developed using the panel of radiation-responsive blood plasma proteomic biomarkers that were negligibly affected by wounding (data not shown). The robustness of this algorithm to accurately predicted the dose for combined injury samples was evaluated. Mice received 0 or 6 Gy 60Co gamma rays with (labeled I) or without (labeled N) skin wounding. Figure 6 shows the results from this study and illustrates individual animal dose predictions. Points within the dashed lines that showed acceptable accuracy criteria are found within the dashed lines shown in Figure 6. These findings illustrate that the selection of appropriate biomarkers, which are negligibly responsive to wounding, can minimize potential confounding effects of combined wound injury when using plasma proteomic biomarkers for dose assessment using a mouse radiation combined injury (wounding) model.

The time window between radiation and wounding may affect the proteomic biomarker response. Further studies including an investigation of the possibility of a time-windows confounder need to be performed to evaluate the utility of a panel of proteomic biomarkers for dose assessment involving combined injury.

## Material and methods

### Radiation model and proteomic analysis

Female B6D2F1/J mice radiation vs CI model and radiation dosimetry was performed as described in Kiang et al. (2010b) except mice were double-loaded in mouse irradiation Plexiglass boxes and exposed to ^60^Co gamma rays doses of 0, 1.5, 3, 6, 10, 12, and 14 Gy at 0.6 Gy min^−1^. Wound trauma involving a dorsal puncture over 15% total body surface area in the mouse CI model was performed 1–2 hr after irradiation. Blood biosampling was performed at 0, 1, 2, 3, 5, and 7 d after irradiation. The number of mice per dose and time point was 8. A panel of candidate radiation-responsive plasma protein biomarkers were measured using the Meso Scale Diagnostic (MSD) MULTI-ARRAY electro-chemiluminescence-detection technology, which exhibited high sensitivity and dynamic range capabilities, as previously described (Debad et al. 2004). Assays were developed as multiplex panels in 16-spot MULTIARRAY 96-well plates and analyzed on an MSD PR2 Model 1800 plate reader as previously described (Sigal et al. 2013).

### Dose prediction algorithm

Dose prediction algorithms were developed as previously described (Sigal et al. 2013). Briefly, plot average response surface in dose and time for each biomarker was developed to predict the dose for an individual sample. The target-specific algorithms selected the dose that best matches the response surface for all of the selected biomarkers of a panel in the model. All panels were evaluated and the best panel that predicted doses for ~94% of samples were selected using a root mean square error analysis.

### Statistical analysis

Data analysis and graphs were done using MS excel (Microsoft Inc.). Comparisons of results for radiation vs radiation and wounding were evaluated using a two-sample T-test using an online tool (Statistics Kingdom) accessible at the website https://www.statskingdom.com/140MeanT2eq.html.

## Summary

The effects of combined radiation with wounding, burns, hemorrhage, or infection represent significant confounders in understanding the resultant radio-response (in particular, organ impairment and survival as well as delayed outcomes) and the development of preparation for medical response both in the assessment and medical management of injuries. Efforts to investigate the CI murine model established at AFRRI have been reviewed with a focus on characterizing the early-phase molecular responses, promising results on the use of single and combined medical countermeasures and preliminary findings on a proposed use of plasma proteomic biomarker panel, negligibly influenced by the combined insults of radiation and wounding, for radiation dose assessment. Follow-on studies are needed to further characterize the effects of CI on molecular mechanisms underlying RI, and validate both uses of medical countermeasures and radiation diagnosis following CI.

## Supplementary Material

Supplementary Table 1

## Figures and Tables

**Table 1.** Percentage of victims exposed to atomic bombs with numbers of injury (Joint Commission of USA and Japanese Physicians collected from victims exposed to 15 KT and 25 KT fission Devices 1946).

	Little boy-U235 (*N* = 5185)	Fat man-Pu239 (*N* = 4107)
Single injury	60.5%	57.5%
Two injuries	34.5%	37.1%
Three injuries	5.0%	5.2%

**Table 2.** Changes in molecules found in a mouse model of radiation followed by wounding, burning, or hemorrhage trauma.

Molecule	RI + WND	RI + Burn	RI + Hemo	Molecule	RI + WND	RI + burn	RI + Hemo
NF-*κ*B	↑	ND	↑	MCP-1	↑	ND	↑
STAT3	↑	ND	↑	MIP-1*α*/*β*	↑	ND	↑
iNOS	↑	ND	↑	IFN-γ	—	ND	—
Claudin-2	↑	ND	ND	Rantes	—	ND	—
IL-1*β*	↑	ND	↑	AKT	↓	ND	—
IL-3	—	ND	↑	MAPK	↑	ND	↑
IL-6	↑	ND	↑	CRP	↑	↓	↓
KC (i.e., IL-8)	↑	ND	↑	C3	↑	↑	↑
IL-10	↑	ND	↑	Corticosterone	↑	↑	↑
IL-12p70	↑	ND	↑	IgM	↓	↓	↑
IL-13	↑	ND	↑	PGE2	↑	↑	—
IL-18	↑	ND	↑	ATP	↑	↑	↑
TNF-*α*	↑	ND	↑	miR-34	↑	ND	↑
G-CSF	↑	ND	—	Caspase-3	↑	ND	↑
GM-CSF	↑	ND	↑	Flt-3L	↑	ND	↑
Exotaxin	↑	ND	↑	Citrulline	↓	ND	ND

NF-*κ*B: Nuclear factor-keppa B; STAT3: Signal transducer and activator of transcription 3; iNOS: inducible nitric oxide synthase; IL-interlukin; KC: Keratinocyte chemoattractant; TNF: Tumor necrosis factor; G-CSF: Granulocyte-colony stimulating factor; GM-CSF: Granulocyte-Macrophage-colony stimulating factor; MCP-1: Monocyte chemoattractant protein-1; MIP-1: Macrophage inflammatory protein; IFN: Interferon; AKT: Protein kinase B; MAPK: Mitogen-activated protein kinase; CRP: C-reactive protein; C3: Complement component 3; IgM: Immunoglobulin M; PGE2: Prostaglandin E2; ATP: Adrine trisphophate; miR: MicroRNA; Flt-3L: Fms-like tyrosine kinase 3 ligand; RI: Radiation Injury; WND: Wound; Hemo: Hemorrhage; ↑: Increase; ND: Not done; ↓: Decrease;—:Not different from radiation alone.

**Table 3.** Drugs that improve survival after lethal irradiation alone (RI) or combined radiation with wound or burn trauma (CI).

Single drug	Species	Rad. dose	Admin. route	Admin. doses	Improvement		References
					RI	CI	
Neupogen (G-CSF)	B6D2F1 female mice	9.5 Gy ^60^Co	SC	10 *μ*g/kg; once daily +1–14d	Yes for survival	No for survival	Kiang et al. Oxid Med Cell Longev 2014a; 2014, 481392.
Neulasta (pegylated G-CSF)	B6D2F1 female mice	9.5 Gy ^60^Co	SC	1 mg/kg; once +1d, +8d, +15d	Yes for survival	No for survival	Kiang et al. Oxid Med Cell Longev 2014a; 2014, 481392.
Alx4100TPO	B6D2F1 female mice	9.5 Gy ^60^Co	SC	1 mg/kg, once +1d	No for survival	Yes for survival	Kiang et al. Mediators of Inflamm 2017; 2017, 7582079.
Mouse MSCs	B6D2F1 female mice	9.25–9.75 Gy ^60^Co	IV	3×10^6^ cells/mouse +1d	ND	Yes for survival	Kiang and Gorbunov, J Cell Sci Ther 2014; 5, 6.
Mouse Ghrelin	B6D2F1 female mice	9.5 Gy ^60^Co	IV	113 *μ*g/kg; once +1d, +2d, +3d	NO for survival	Yes for survival	Kiang et al Oxid Med Cell Longe 2014b; 2014, 215858; Cell Biosci 2018; 8, 27 and 2020; 10, 63.
Human Ghrelin	Sprague-Dowley male rats	10 Gy gamma	SC	20 nmol/rat	Yes for survival	ND for CI	Wang et al. PLoS ONE 2015; 10(2), e0118213.
Ciprofloxacin	B6D2F1 female mice	9.25 Gy ^60^Co	PO	90 mg/kg, po once, 2 h and 1d–21d	No for survival	Yes for survival	Fukumoto et al. PLOS ONE 2014; 9(2), e90448
Gentamicin	C3H/HeN-J female mice	Dose–response; 67% neutron + 33% gamma	Topical	4hr after and +1d–+9d	NA	Yes for survival with DRF = 1.36 (mixed–field)	Ledney and Elliott, Health Phys 2010; 98(2), 145–52
Silvadene	C3H/HeN-J female mice	Dose–response; 67% neutron + 33% gamma	Topical	4hr after and +1d–+9d	NA	Yes for survival with DRF = 1.16 (mixed–field)	Ledney and Elliott, Health Phys 2010; 98(2), 145–152
WR-151327	C3H/HeN-J female mice	Dose–response; 67% neutron + 33% gamma or 100% gamma alone	IP	200 mg/kg, 30min before irradiation	Yes for survival with DRF = 1.53 (gamma) and 1.31 (mixed–field)	Yes for survival with DRF = 1.51 (gamma) and 1.22 (mixed–field)	Ledney and Elliott, Health Phys 2010; 98(2), 145–152
Bone marrow transplantation	B6D2F1 female mice	10 Gy ^60^Co	IV	Given after RI or CI. Time was not known. For CI, needed 10X more than RI	Yes for survival	Yes for survival	Ledney and Elliott, Health Phys 2010; 98(2), 145–152

Rad.: radiation; Admin.: administration; SC: subcutaneous; PO: per os (i.e., oral feed); IV: intravenous; IP: intrapreural; RI: radiation injury; CI: combined radiation injury; ND: not done; MSC: mesenchymal stem cells; TPO: thrombopoietin; DRF: dose reduction factor.

**Table 4.** Combined drugs that improve survival after lethal irradiation alone (RI) or combined radiation with wound or burn trauma (CI).

2 drugs	Species	Rad. dose	Admin. route	Admin. doses	Improvement		References
					RI	CI	
Neulasta + Alxn4100TPO	B6D2F1 female mice	9.5 Gy ^60^Co	SC; SC	1mg/kg sc + 1d, +8d, +15d; 1 mg/kg sc, +1d	Yes for survival	Yes for survival	Kiang et al. Radiat Res 2017; 188, 476–490.
Neulasta + Ghrelin	B6D2F1 female mice	9.5 Gy ^60^Co	SC; SC	1mg/kg sc + 1d, +8d, +15d; 113 *μ*g/kg; once +1d, +2d, +3d*μ*	Yes for survival	No for survival	Kiang et al. Front Pharmacol 2021; 2021:628018
Neulasta + Ciprofloxacin	B6D2F1 female mice	9.5 Gy Co-60	SC; PO	1mg/kg sc + 1d, +8d, +15d; 90 mg/kg, po once, +2 hr, +1d–+21d*μ*	Yes for survival	No for survival	Kiang et al. in preparation
Neulasta + L-Citrulline	B6D2F1 female mice	9.5 Gy ^60^Co	SC; PO	1mg/kg sc +1d, +8d, +15d; 1 g/kg; po once +1d-21d	Yes for survival	almost for survival	Wang et al. Radiat Res 2021; 196, 113–127
S-TDCM + Gentamicin	C3H/HeN-J female mice	8Gy ^60^Co	IP	200 mg/kg, immediately after irradiation	No for survival	Yes for survival	Ledny and Elliott, Health Phys 2010; 98(2), 145–152

Rad.: radiation; Admin.: administration; SC: subcutaneous; PO: per os (i.e., oral feed); IP: intrapreural; RI: radiation injury; CI: combined radiation injury.

## Data Availability

Data presented in this review are available in it.
