# Supplementary material for: Combined radiation injury and its impacts on radiation countermeasures and biodosimetry
Source: Int J Radiat Biol. Author manuscript; Available in PMC 2024 Mar 22. (PMC10947598; doi:10.1080/09553002.2023.2188933)
Supplement: Supplementary Table 1 [file NIHMS1973684-supplement-Supplementary_Table_1.docx]

**Supplemental Table 1. Statistical analysis of data presented in Figures 3-5.**

**Figure 3. SAA after combined injury**

| Two sample t-test | | | | |  |  |  |  |  |  |  |  |
| --- | --- | --- | --- | --- | --- | --- | --- | --- | --- | --- | --- | --- |
| Dose, Gy | 0 |  |  |  |  |  |  |  | | | |  |
| Time |  | p-value | T-statistic | Effect size |  | X1 | SE | X2 | SE |  | AVG | SE |
|  |  |  |  |  |  |  |  |  |  |  |  |  |
| 0 | Reject Ho | 1.35E-09 | -13.9312 | 6.97 |  | 1106 | 271.3695 | 28976 | 3353.463 |  | 0.038182 | 0.010355 |
| 1 | Reject Ho | 4.05E-07 | -8.8671 | 4.43 |  | 1204 | 299.7139 | 80698 | 15074.34 |  | 0.014924 | 0.004644 |
| 2 | Reject Ho | 0.0000858 | -5.4485 | 2.72 |  | 959 | 131.9777 | 88517 | 27026.46 |  | 0.010839 | 0.00363 |
| 3 | Reject Ho | 0.000001073 | -8.1687 | 4.08 |  | 989 | 91.88337 | 99880 | 20359.6 |  | 0.009906 | 0.002219 |
| 5 | Reject Ho | 0.000009626 | -6.73 | 3.36 |  | 1159 | 305.3844 | 71550 | 17587.62 |  | 0.016205 | 0.005838 |
| 7 | Reject Ho | 0.00183 | -3.8325 | 1.92 |  | 1211 | 181.0367 | 20913 | 8643.507 |  | 0.057915 | 0.025454 |
|  |  |  |  |  |  |  |  |  |  |  |  |  |
| Reject Ho = average of 1st group is not equal to average of group 2 | | | | | | | | | | | | |
|  |  |  |  |  |  |  |  |  |  |  |  |  |
| Dose, Gy | 3 |  |  |  |  |  |  |  |  |  |  |  |
| Time |  | p-value | T-statistic | Effect size |  |  |  |  |  |  |  |  |
|  |  |  |  |  |  |  |  |  |  |  |  |  |
| 0 | Reject Ho | 2.17E-02 | -2.5835 | 1.29 |  | 1211 | 247.467 | 34241 | 3353.457 |  | 0.035377 | 0.008015 |
| 1 | Reject Ho | 1.58E-04 | -5.1142 | 2.56 |  | 20803 | 7992.281 | 59754 | 15074.32 |  | 0.348147 | 0.160011 |
| 2 | Reject Ho | 5.82E-07 | -8.1115 | 4.06 |  | 3546 | 659.3822 | 90916 | 27026.41 |  | 0.039007 | 0.013677 |
| 3 | Reject Ho | 3.06E-11 | -17.5842 | 8.79 |  | 875 | 62.80915 | 88785 | 20359.57 |  | 0.009853 | 0.002368 |
| 5 | Reject Ho | 1.92E-08 | -10.7369 | 5.37 |  | 1312 | 523.636 | 69572 | 17587.59 |  | 0.018861 | 0.00891 |
| 7 | Reject Ho | 1.17E-03 | -3.7079 | 1.85 |  | 916 | 79.64524 | 14037 | 8643.492 |  | 0.065273 | 0.04059 |
|  |  |  |  |  |  |  |  |  |  |  |  |  |
| Reject Ho = average of 1st group is not equal to average of group 2 | | | | | | | | | | | | |
|  |  |  |  |  |  |  |  |  |  |  |  |  |
| Dose, Gy | 6 |  |  |  |  |  |  |  |  |  |  |  |
| Time |  | p-value | T-statistic | Effect size |  |  |  |  |  |  |  |  |
|  |  |  |  |  |  |  |  |  |  |  |  |  |
| 0 | Reject Ho | 4.86E-04 | -4.5141 | 2.26 |  | 1433 | 248.3404 | 31517 | 3353.457 |  | 0.045483 | 0.009247 |
| 1 | Reject Ho | 6.17E-03 | -3.2203 | 1.61 |  | 36966 | 8020.488 | 65646 | 15074.32 |  | 0.563112 | 0.177898 |
| 2 | Reject Ho | 6.35E-11 | -17.5333 | 8.77 |  | 6880 | 661.7093 | 104885 | 27026.41 |  | 0.065597 | 0.018042 |
| 3 | Reject Ho | 1.26E-07 | -9.7587 | 4.88 |  | 1099 | 63.03082 | 94029 | 20359.57 |  | 0.01169 | 0.002618 |
| 5 | Reject Ho | 4.44E-04 | -4.5616 | 2.28 |  | 985 | 525.4841 | 145862 | 17587.59 |  | 0.006751 | 0.003693 |
| 7 | Reject Ho | 3.38E-07 | -9.0018 | 4.5 |  | 1510 | 79.92633 | 28982 | 8643.492 |  | 0.052096 | 0.01578 |
|  |  |  |  |  |  |  |  |  |  |  |  |  |
| Reject Ho = average of 1st group is not equal to average of group 2 | | | | | | | | | | | | |
|  |  |  |  |  |  |  |  |  |  |  |  |  |
| Dose, Gy | 10 |  |  |  |  |  |  |  |  |  |  |  |
| Time |  | p-value | T-statistic | Effect size |  |  |  |  |  |  |  |  |
|  |  |  |  |  |  |  |  |  |  |  |  |  |
| 0 | Reject Ho | 1.50E-08 | -11.5644 | 5.78 |  | 3472 | 248.3417 | 28076 | 3353.457 |  | 0.123664 | 0.017217 |
| 1 | Reject Ho | 5.17E-04 | -4.482 | 2.24 |  | 39029 | 8020.531 | 73384 | 15074.32 |  | 0.531847 | 0.154535 |
| 2 | Reject Ho | 9.19E-08 | -10.0131 | 5.01 |  | 7657 | 661.7128 | 108447 | 27026.41 |  | 0.070604 | 0.018623 |
| 3 | Reject Ho | 3.70E-04 | -4.657 | 2.33 |  | 3311 | 63.03116 | 132247 | 20359.57 |  | 0.025038 | 0.003884 |
| 5 | Reject Ho | 1.77E-12 | -22.835 | 11.42 |  | 1072 | 525.4869 | 116509 | 17587.59 |  | 0.009204 | 0.004719 |
| 7 | Reject Ho | 4.32E-12 | -21.3894 | 10.69 |  | 1217 | 79.92676 | 134934 | 8643.492 |  | 0.009023 | 0.000828 |
|  |  |  |  |  |  |  |  |  |  |  |  |  |
| Reject Ho = average of 1st group is not equal to average of group 2 | | | | | | | | | | | | |

**Figure 4. Flrt-3 ligand after combined injury.**

| Dose, Gy | 0 | | | |  |  |  |  |  |  |  |  |  |
| --- | --- | --- | --- | --- | --- | --- | --- | --- | --- | --- | --- | --- | --- |
| Time |  | p-value | T-statistic | Effect size |  | X1 | SE | X2 | SE |  | AVG | SE |  |
|  |  |  |  |  |  |  |  |  |  |  |  |  |  |
| 0 | Ho cannot be rejected | 9.35E-01 | -0.9351 | 0.041 |  | 597 | 59.30523 | 601 | 55.48443 |  | 0.993 | 0.13466 |  |
| 1 | Reject Ho | 4.44E-02 | 2.208 | 1.1 |  | 580 | 73.77086 | 459 | 55.08903 |  | 1.264 | 0.221027 |  |
| 2 | Ho cannot be rejected | 0.1184 | 1.6638 | 0.83 |  | 597 | 55.33008 | 510 | 68.10629 |  | 1.172 | 0.190568 |  |
| 3 | Reject Ho | 0.005005 | 3.3252 | 1.66 |  | 700 | 70.47487 | 526 | 52.16264 |  | 1.333 | 0.188303 |  |
| 5 | Ho cannot be rejected | 0.1409 | -1.5606 | 0.78 |  | 599 | 53.14237 | 691 | 83.88911 |  | 0.867 | 0.130244 |  |
| 7 | Ho cannot be rejected | 0.8365 | 0.2103 | 0.11 |  | 633 | 56.47772 | 624 | 44.85102 |  | 1.014 | 0.116198 |  |
|  |  |  |  |  |  |  |  |  |  |  |  |  |  |
| Reject Ho = average of 1st group is not equal to average of group 2 | | | | | | | | | | | | |  |
|  |  |  |  |  |  |  |  |  |  |  |  |  |  |
| Dose, Gy | 3 | | | |  |  |  |  |  |  |  |  |  |
| Time |  | p-value | T-statistic | Effect size |  |  |  |  |  |  |  |  |  |
|  |  |  |  |  |  |  |  |  |  |  |  |  |  |
| 0 | Ho cannot be rejected | 8.86E-02 | 1.83 | 0.92 |  | 616 | 72.52119 | 523 | 45.13384 |  | 1.178 | 0.171894 |  |
| 1 | Reject Ho | 6.31E-04 | 4.3777 | 2.19 |  | 1220 | 140.9734 | 818 | 61.89705 |  | 1.491 | 0.205945 |  |
| 2 | Reject Ho | 2.75E-04 | 4.8144 | 2.41 |  | 1923 | 161.2234 | 1240 | 175.533 |  | 1.551 | 0.255059 |  |
| 3 | Reject Ho | 3.15E-05 | 6.0194 | 3.01 |  | 2194 | 154.0776 | 1492 | 120.9365 |  | 1.470 | 0.157677 |  |
| 5 | Ho cannot be rejected | 1.27E-01 | -1.6215 | 0.81 |  | 1414 | 173.0311 | 1637 | 152.5448 |  | 0.864 | 0.132914 |  |
| 7 | Ho cannot be rejected | 3.70E-01 | -0.9257 | 0.46 |  | 999 | 145.3074 | 1123 | 171.5472 |  | 0.890 | 0.187718 |  |
|  |  |  |  |  |  |  |  |  |  |  |  |  |  |
| Reject Ho = average of 1st group is not equal to average of group 2 | | | | | | | | | | | | |  |
|  |  |  |  |  |  |  |  |  |  |  |  |  |  |
| Dose, Gy | 6 | | | |  |  |  |  |  |  |  |  |  |
| Time |  | p-value | T-statistic | Effect size |  |  |  |  |  |  |  |  |  |
|  |  |  |  |  |  |  |  |  |  |  |  |  |  |
| 0 | Reject Ho | 2.20E-03 | 3.7401 | 1.87 |  | 784 | 56.30594 | 645 | 26.65309 |  | 1.214 | 0.100631 |  |
| 1 | Reject Ho | 3.18E-04 | 4.7381 | 2.37 |  | 1642 | 145.0027 | 1126 | 112.0291 |  | 1.459 | 0.194076 |  |
| 2 | Reject Ho | 1.66E-06 | 7.8705 | 3.94 |  | 3579 | 367.259 | 1812 | 86.62261 |  | 1.975 | 0.223556 |  |
| 3 | Reject Ho | 8.39E-05 | 5.4606 | 2.73 |  | 4847 | 399.3237 | 2948 | 271.1592 |  | 1.644 | 0.203041 |  |
| 5 | Reject Ho | 6.13E-05 | 5.6368 | 2.82 |  | 5982 | 561.975 | 3841 | 303.7411 |  | 1.557 | 0.191267 |  |
| 7 | Ho cannot be rejected | 5.72E-01 | -0.5783 | 0.29 |  | 5081 | 357.0794 | 5258 | 370.2508 |  | 0.966 | 0.096131 |  |
|  |  |  |  |  |  |  |  |  |  |  |  |  |  |
| Reject Ho = average of 1st group is not equal to average of group 2 | | | | | | | | | | | | |  |
|  |  |  |  |  |  |  |  |  |  |  |  |  |  |
| Dose, Gy | 10 | | | |  |  |  |  |  |  |  |  |  |
| Time |  | p-value | T-statistic | Effect size |  |  |  |  |  |  |  |  |  |
|  |  |  |  |  |  |  |  |  |  |  |  |  |  |
| 0 | Reject Ho | 2.61E-03 | 3.6527 | 1.83 |  | 867 | 62.41659 | 685 | 55.69008 |  | 1.265 | 0.137344 |  |
| 1 | Reject Ho | 8.29E-06 | 6.8228 | 3.41 |  | 2274 | 79.44333 | 1765 | 100.591 |  | 1.288 | 0.086116 |  |
| 2 | Reject Ho | 8.02E-07 | 8.3731 | 4.19 |  | 4831 | 302.4639 | 2770 | 282.3762 |  | 1.744 | 0.208705 |  |
| 3 | Reject Ho | 4.07E-04 | 4.6066 | 2.3 |  | 5148 | 385.1524 | 3546 | 439.7896 |  | 1.452 | 0.21026 |  |
| 5 | Reject Ho | 1.35E-05 | 6.5222 | 3.26 |  | 7397 | 505.1872 | 4849 | 378.1645 |  | 1.525 | 0.158136 |  |
| 7 | Reject Ho | 3.05E-05 | 6.038 | 3.02 |  | 7298 | 583.0343 | 4974 | 280.8137 |  | 1.467 | 0.143512 |  |
|  |  |  |  |  |  |  |  |  |  |  |  |  |  |
| Reject Ho = average of 1st group is not equal to average of group 2 | | | | | | | | | | | | |  |

**Figure 5. CD27 after combined injury**

| Dose,Gy | 0 | | | |  |  |  |  |  |  |  |  |  |  |  |  |  |  |  |  |  |  |  |  |  |
| --- | --- | --- | --- | --- | --- | --- | --- | --- | --- | --- | --- | --- | --- | --- | --- | --- | --- | --- | --- | --- | --- | --- | --- | --- | --- |
| Time |  | p-value | T-statistic | Effect size |  | X1 | SE | X2 | SE |  | AVG | SE |  |  |  |  |  |  |  |  |  |  |  |  |  |
|  |  |  |  |  |  |  |  |  |  |  | #DIV/0! | #DIV/0! |  |  |  |  |  |  |  |  |  |  |  |  |  |
| 0 | Ho cannot be rejected | 9.17E-01 | 0.1058 | 0.053 |  | 2417 | 543.9344 | 2353 | 859.6244 |  | 1.027552 | 0.440946 |  |  |  |  |  |  |  |  |  |  |  |  |  |
| 1 | Ho cannot be rejected | 2.53E-01 | 1.1911 | 0.6 |  | 1606 | 215.3423 | 1296 | 381.0591 |  | 1.238666 | 0.400243 |  |  |  |  |  |  |  |  |  |  |  |  |  |
| 2 | Ho cannot be rejected | 0.07474 | 1.9254 | 0.96 |  | 1460 | 181.7497 | 1130 | 223.6266 |  | 1.292241 | 0.302219 |  |  |  |  |  |  |  |  |  |  |  |  |  |
| 3 | Reject Ho | 0.009717 | 2.9913 | 1.5 |  | 1673 | 323.6302 | 1017 | 177.1513 |  | 1.644723 | 0.428213 |  |  |  |  |  |  |  |  |  |  |  |  |  |
| 5 | Ho cannot be rejected | 0.5402 | 0.6276 | 0.31 |  | 1546 | 239.7603 | 1373 | 396.6059 |  | 1.125854 | 0.369104 |  |  |  |  |  |  |  |  |  |  |  |  |  |
| 7 | Ho cannot be rejected | 0.1268 | 1.6235 | 0.81 |  | 1411 | 132.5859 | 1232 | 129.3573 |  | 1.145961 | 0.16147 |  |  |  |  |  |  |  |  |  |  |  |  |  |
|  |  |  |  |  |  |  |  |  |  |  |  |  |  |  |  |  |  |  |  |  |  |  |  |  |  |
| Reject Ho = average of 1st group is not equal to average of group 2 | | | | | | | | | | | | |  |  |  |  |  |  |  |  |  |  |  |  |  |
|  |  |  |  |  |  |  |  |  |  |  |  |  |  |  |  |  |  |  |  |  |  |  |  |  |  |
| Dose, Gy | 3 | | | |  |  |  |  |  |  |  |  |  |  |  |  |  |  |  |  |  |  |  |  |  |
| Time |  | p-value | T-statistic | Effect size |  |  |  |  |  |  |  |  |  |  |  |  |  |  |  |  |  |  |  |  |  |
|  |  |  |  |  |  |  |  |  |  |  |  |  |  |  |  |  |  |  |  |  |  |  |  |  |  |
| 0 | Ho cannot be rejected | 2.00E-01 | 1.3458 | 0.67 |  | 3223 | 480.8212 | 2699 | 443.2509 |  | 1.194337 | 0.265019 |  |  |  |  |  |  |  |  |  |  |  |  |  |
| 1 | Ho cannot be rejected | 4.07E-01 | 0.8553 | 0.43 |  | 616 | 130.0669 | 283 | 51.02622 |  | 2.174438 | 0.603593 |  |  |  |  |  |  |  |  |  |  |  |  |  |
| 2 | Ho cannot be rejected | 1.68E-01 | 1.4529 | 0.73 |  | 255 | 33.77466 | 202 | 51.16721 |  | 1.260271 | 0.359856 |  |  |  |  |  |  |  |  |  |  |  |  |  |
| 3 | Ho cannot be rejected | 9.16E-02 | 1.811 | 0.91 |  | 253 | 41.2581 | 202 | 22.48441 |  | 1.250813 | 0.246962 |  |  |  |  |  |  |  |  |  |  |  |  |  |
| 5 | Reject Ho | 1.51E-02 | 2.7688 | 1.38 |  | 463 | 55.84193 | 350 | 51.71485 |  | 1.323121 | 0.252175 |  |  |  |  |  |  |  |  |  |  |  |  |  |
| 7 | Ho cannot be rejected | 1.54E-01 | 1.5091 | 0.75 |  | 716 | 99.77554 | 575 | 121.0549 |  | 1.245514 | 0.314583 |  |  |  |  |  |  |  |  |  |  |  |  |  |
|  |  |  |  |  |  |  |  |  |  |  |  |  |  |  |  |  |  |  |  |  |  |  |  |  |  |
| Reject Ho = average of 1st group is not equal to average of group 2 | | | | | | | | | | | | |  |  |  |  |  |  |  |  |  |  |  |  |  |
|  |  |  |  |  |  |  |  |  |  |  |  |  |  |  |  |  |  |  |  |  |  |  |  |  |  |
| Dose, Gy | 6 | | | |  |  |  |  |  |  |  |  |  |  |  |  |  |  |  |  |  |  |  |  |  |
| Time |  | p-value | T-statistic | Effect size |  |  |  |  |  |  |  |  |  |  |  |  |  |  |  |  |  |  |  |  |  |
|  |  |  |  |  |  |  |  |  |  |  |  |  |  |  |  |  |  |  |  |  |  |  |  |  |  |
| 0 | Ho cannot be rejected | 2.74E-01 | 1.1386 | 0.57 |  | 4338 | 737.3851 | 3715 | 550.85 |  | 1.167703 | 0.263399 |  |  |  |  |  |  |  |  |  |  |  |  |  |
| 1 | Reject Ho | 3.13E-03 | 3.5608 | 1.78 |  | 382 | 80.4665 | 198 | 33.28333 |  | 1.93477 | 0.521794 |  |  |  |  |  |  |  |  |  |  |  |  |  |
| 2 | Reject Ho | 2.90E-03 | 3.6006 | 1.8 |  | 145 | 24.67293 | 78 | 19.65625 |  | 1.850684 | 0.562559 |  |  |  |  |  |  |  |  |  |  |  |  |  |
| 3 | Reject Ho | 9.23E-04 | 4.1819 | 2.09 |  | 115 | 18.52498 | 62 | 10.65507 |  | 1.863301 | 0.439903 |  |  |  |  |  |  |  |  |  |  |  |  |  |
| 5 | Reject Ho | 3.60E-03 | 3.4913 | 1.75 |  | 152 | 24.78356 | 89 | 17.52761 |  | 1.698623 | 0.434026 |  |  |  |  |  |  |  |  |  |  |  |  |  |
| 7 | Reject Ho | 6.90E-06 | 6.9377 | 3.47 |  | 457 | 47.82648 | 176 | 48.50011 |  | 2.604411 | 0.769588 |  |  |  |  |  |  |  |  |  |  |  |  |  |
|  |  |  |  |  |  |  |  |  |  |  |  |  |  |  |  |  |  |  |  |  |  |  |  |  |  |
| Reject Ho = average of 1st group is not equal to average of group 2 | | | | | | | | | | | | |  |  |  |  |  |  |  |  |  |  |  |  |  |
|  |  |  |  |  |  |  |  |  |  |  |  |  |  |  |  |  |  |  |  |  |  |  |  |  |  |
| Dose, Gy | 10 | | | |  |  |  |  |  |  |  |  |  |  |  |  |  |  |  |  |  |  |  |  |  |
| Time |  | p-value | T-statistic | Effect size |  |  |  |  |  |  |  |  |  |  |  |  |  |  |  |  |  |  |  |  |  |
|  |  |  |  |  |  |  |  |  |  |  |  |  |  |  |  |  |  |  |  |  |  |  |  |  |  |
| 0 | Ho cannot be rejected | 8.50E-01 | 0.1923 | 0.096 |  | 3709 | 598.838 | 3616 | 830.1572 |  | 1.025653 | 0.287856 |  |  |  |  |  |  |  |  |  |  |  |  |  |
| 1 | Reject Ho | 1.31E-02 | 2.8388 | 1.42 |  | 186 | 31.96457 | 123 | 18.84155 |  | 1.511693 | 0.348667 |  |  |  |  |  |  |  |  |  |  |  |  |  |
| 2 | Reject Ho | 1.13E-02 | 2.9134 | 1.46 |  | 83 | 18.36448 | 45 | 11.77021 |  | 1.838349 | 0.631749 |  |  |  |  |  |  |  |  |  |  |  |  |  |
| 3 | Reject Ho | 3.37E-04 | 4.7068 | 4.7068 |  | 70 | 10.56042 | 34 | 7.12097 |  | 2.069053 | 0.539152 |  |  |  |  |  |  |  |  |  |  |  |  |  |
| 5 | Reject Ho | 8.99E-03 | 3.0304 | 1.52 |  | 83 | 20.36713 | 44 | 7.976 |  | 1.86262 | 0.566546 |  |  |  |  |  |  |  |  |  |  |  |  |  |
| 7 | Reject Ho | 1.35E-02 | 2.8252 | 1.41 |  | 84 | 22.32788 | 42 | 10.71805 |  | 2.027153 | 0.749364 |  |  |  |  |  |  |  |  |  |  |  |  |  |
|  |  |  |  |  |  |  |  |  |  |  |  |  |  |  |  |  |  |  |  |  |  |  |  |  |  |
| Reject Ho = average of 1st group is not equal to average of group 2 | | | | | | | | | | | | |  |  |  |  |  |  |  |  |  |  |  |  |  |
|  | | | | | | | | | | | | |  |  |  |  |  |  |  |  |  |  |  |  |  |
| <https://www.statskingdom.com/140MeanT2eq.html> | | | | | | | | | | | | |  |  |  |  |  |  |  |  |  |  |  |  |  |
|  | | | | | | | | | | | | |  |  |  |  |  |  |  |  |  |  |  |  |  |
|  | | | | | | | | | | | | |  |  |  |  |  |  |  |  |  |  |  |  |  |
| Since p-value > α, H0 cannot be rejected. | | | | | | | | | | | | |  |  |  |  |  |  |  |  |  |  |  |  |  |
| The average of **Group-1's** population is assumed to be **equal to**the average of **Group-2's** population. | | | | | | | | | | | | |  |  |  |  |  |  |  |  |  |  |  |  |  |
| In other words, the difference between the sample average of **Group-1** and **Group-2** is not big enough to be statistically significant. | | | | | | | | | | | | |  |  |  |  |  |  |  |  |  |  |  |  |  |
